# Results from the Survey of Antibiotic Resistance (SOAR) 2018–21 in Pakistan: data based on CLSI, EUCAST (dose-specific) and pharmacokinetic/pharmacodynamic (PK/PD) breakpoints

**DOI:** 10.1093/jac/dkaf288

**Published:** 2025-11-24

**Authors:** Didem Torumkuney, Summiya Nizamuddin, Ian Morrissey, Rendani Manenzhe, Anand Manoharan

**Affiliations:** Infectious Diseases Research Unit, GSK, London, UK; Department of Microbiology, Shaukat Khanum Memorial Cancer Hospital and Research Centre, Lahore, Pakistan; Antimicrobial Focus Ltd., Sawbridgeworth, UK; Infectious Diseases Research Unit, GSK, Gauteng, South Africa; Infectious Diseases Medical & Scientific Affairs, GSK, Mumbai, India

## Abstract

**Objectives:**

To determine the antibiotic susceptibility of *Streptococcus pneumoniae* and *Haemophilus influenzae* isolates from community-acquired respiratory tract infections (CA-RTIs) collected in 2018–21 from Pakistan.

**Methods:**

MICs were determined by CLSI broth microdilution; susceptibility data were interpreted using CLSI, EUCAST and pharmacokinetic/pharmacodynamic (PK/PD) breakpoints.

**Results:**

*S. pneumoniae* (*n* = 57) and *H. influenzae* (*n* = 67) were collected from the Shaukat Khanum Memorial Cancer Hospital and Research Centre, Lahore, Pakistan. The proportion of penicillin-susceptible pneumococci was 28.1% by CLSI oral/EUCAST low-dose breakpoints; 98.3%/98.2% were susceptible by EUCAST high-dose/CLSI intravenous breakpoints. Good activity (≥93.0%) was observed using CLSI or PK/PD breakpoints for amoxicillin, amoxicillin/clavulanic acid, cefotaxime, cefpodoxime, ceftriaxone, cefuroxime, levofloxacin and moxifloxacin. Cefdinir and cefaclor were less active (75.4% and 57.9%, respectively, by CLSI and 61.4% and 19.3%, respectively, by PK/PD). Tetracyclines, macrolides and trimethoprim/sulfamethoxazole were poorly active (5.3%–36.8%). EUCAST high-dose breakpoints indicated similar activity, although susceptibility to cefaclor (0%), cefpodoxime (70.2%) and cefuroxime (68.4%) was lower than by CLSI. Most *H. influenzae* isolates were β-lactamase negative (95.5%). Generally, antibiotic susceptibility was >83.6% by CLSI for all antibiotics except trimethoprim/sulfamethoxazole (13.4%). Susceptibility by EUCAST was similar, except for cefuroxime (oral), with 0% susceptible isolates versus 95.5% by CLSI and 67.2% by PK/PD, fluoroquinolones and macrolides (no EUCAST breakpoint). Macrolide susceptibility by PK/PD breakpoints was low.

**Conclusions:**

Although antimicrobial resistance was observed, many therapeutic options remain to treat *S. pneumoniae* and *H. influenzae* from CA-RTI in Pakistan despite concerns about high levels of inappropriate antibiotic use. Continued surveillance of antibiotic susceptibility is important for guiding empiric therapy of CA-RTIs.

## Introduction

Community-acquired respiratory tract infections (CA-RTIs) are an important world health problem that, if treated inappropriately, or in patients with comorbidities, can result in hospitalization, with a third of patients with community-acquired pneumonia dying within 12 months after being discharged from hospital.^1^ However, age, comorbidities and other underlying risk factors may have influenced the observed mortality rate.^1^ Treatment of CA-RTIs is reliant on empiric antibiotic therapy through the use of national and international guidelines.^2^ Studies have shown that there is a high level of inappropriate antibiotic use in Pakistan, including sales without a prescription and self-medication,^3–5^ and this has been shown to be associated with the development of increasing antimicrobial resistance.^6^

*Streptococcus pneumoniae* and *Haemophilus influenzae* are the major bacteria associated with CA-RTIs.^7,8^ Both pathogens have shown increasing resistance to first-line antibiotics such as penicillin and ampicillin.^9,10^ As rates of resistance vary over time and from country to country, up-to-date surveillance data are essential to guide local antibiotic policies.^11^

The Survey of Antibiotic Resistance (SOAR), an international antibiotic resistance surveillance study, focuses on key respiratory pathogens that cause community-acquired infections and has been running since 2002 in the Middle East, Africa, Latin America, Asia-Pacific, Europe and the Commonwealth of Independent States countries.^12^ For this study, recent SOAR data from hospitals in Pakistan have been analysed to provide a picture of the current state of antibiotic susceptibility of *S. pneumoniae* and *H. influenzae* associated with CA-RTIs.

## Materials and methods

### Ethics

SOAR studies are not human subject studies. During the study, only microorganisms were examined.

### Clinical isolates

Isolates of *H. influenzae* and *S. pneumoniae* from CA-RTIs (isolated within 48 h of hospitalization) were collected between 2018 and 2021 from the Shaukat Khanum Memorial Cancer Hospital, Lahore, Pakistan, and sent to a central laboratory (IHMA Europe, Monthey, Switzerland), where they were sub-cultured and re-identified. *H. influenzae* were re-identified by MALDI-TOF MS methodology, and *S. pneumoniae* identity was confirmed by optochin susceptibility and bile solubility. β-lactamase production was determined for each *H. influenzae* isolate by a chromogenic cephalosporin (nitrocefin) disc method. Duplicate isolates from the same patient were not accepted.

### Susceptibility testing

Isolates were evaluated for antibiotic susceptibility using broth microdilution methodology recommended by CLSI.^13^ Amoxicillin, amoxicillin/clavulanic acid (2:1 ratio as per CLSI guidelines^13,14^), amoxicillin/clavulanic acid (fixed clavulanic acid at 2 mg/L as per EUCAST guidelines^15^), azithromycin, cefaclor, cefdinir, cefixime, cefotaxime, cefpodoxime, ceftibuten, ceftriaxone, cefuroxime, clarithromycin, levofloxacin, moxifloxacin, tetracycline and trimethoprim/sulfamethoxazole (1:19 ratio) were tested against both respiratory pathogens. In addition, doxycycline, erythromycin and penicillin were tested against *S. pneumoniae* only, and ampicillin was tested against *H. influenzae* only. Susceptibility to the study drugs was calculated based on CLSI, EUCAST (dose-specific) and pharmacokinetic/pharmacodynamic (PK/PD) breakpoints.^14–16^ These breakpoints are given in Tables 1–3. To fully assess antibiotics where high-dose therapies are available, susceptibility using EUCAST criteria was also calculated by combining percentage susceptible and susceptible, increased exposure into the susceptible category as well as dose-dependent PK/PD breakpoints.^15,16^ The antibiotics with high-dose availability assessed in this way were as follows: amoxicillin (0.75–1 g oral, 3× daily), amoxicillin/clavulanic acid (0.875 g amoxicillin/0.125 g clavulanic acid oral, 3× daily), ampicillin (2 g intravenous [IV], 4× daily), penicillin (2.4 g IV, 2 MU 4–6× daily), ceftriaxone (2 g IV, 2× daily), clarithromycin (0.5 g oral, 2× daily), erythromycin (1 g oral or IV, 4× daily), levofloxacin (0.75 g oral 2× daily or 0.4 g IV 3× daily) and trimethoprim/sulfamethoxazole (0.24 g trimethoprim/1.2 g sulfamethoxazole oral or IV, 2× daily).^15^

**Table 1.** CLSI MIC breakpoints (mg/L) used for *S. pneumoniae* and *H. influenzae* isolates

	*S. pneumoniae*			*H. influenzae*		
Antimicrobial	S	I	R	S	I	R
Amoxicillin	≤2	4	≥8	—	—	—
Amoxicillin/clavulanic acid (2:1)^a^	≤2	4	≥8	≤2	4	≥8
Ampicillin	NT	NT	NT	≤1	2	≥4
Azithromycin	≤0.5	1	≥2	≤4	—	—
Cefaclor	≤1	2	≥4	≤8	16	≥32
Cefdinir	≤0.5	1	≥2	≤1	—	—
Cefixime	—	—	—	≤1	—	—
Cefotaxime (non-meningitis)	≤1	2	≥4	≤2	—	—
Cefpodoxime	≤0.5	1	≥2	≤2	—	—
Ceftibuten	—	—	—	≤2	—	—
Ceftriaxone (non-meningitis)	≤1	2	≥4	≤2	—	—
Cefuroxime^b^	≤1	2	≥4	≤4	8	≥16
Clarithromycin	≤0.25	0.5	≥1	≤8	16	≥32
Doxycycline	≤0.25	0.5	≥1	NT	NT	NT
Erythromycin	≤0.25	0.5	≥1	NT	NT	NT
Levofloxacin	≤2	4	≥8	≤2	—	—
Moxifloxacin	≤1	2	≥4	≤1	—	—
Penicillin (2.4 g 2 MU × 4–6 IV)	≤2	4	≥8	NT	NT	NT
Penicillin (oral)	≤0.06	0.12–1	≥2	NT	NT	NT
Tetracycline	≤1	2	≥4	≤2	4	≥8
Trimethoprim/sulfamethoxazole^c^	≤0.5	1–2	≥4	≤0.5	1–2	≥4

—, not applicable; I, intermediate; NT, not tested; R, resistant; S, susceptible.

^a^Amoxicillin/clavulanic acid was tested at a 2:1 amoxicillin to clavulanic acid ratio; breakpoints are expressed as the amoxicillin component.

^b^Breakpoints used are for cefuroxime axetil (oral).

^c^Trimethoprim/sulfamethoxazole was tested at a 1:19 trimethoprim to sulfamethoxazole ratio; breakpoints are expressed as the trimethoprim component.

**Table 2.** EUCAST (dose-specific) MIC breakpoints (mg/L) used for *S. pneumoniae* and *H. influenzae* isolates

	*S. pneumoniae*		*H. influenzae*	
Antimicrobial^a^	S	R	S	R
Amoxicillin (0.5 g × 3 oral)	≤0.5	>1	≤0.001	>2
Amoxicillin (0.75–1 g × 3 oral)	≤1	>1	≤2	>2
Amoxicillin/clavulanic acid (0.5 g/0.125 g × 3 oral)^b^	≤0.5	>1	≤0.001	>2
Amoxicillin/clavulanic acid (0.875 g/0.125 g × 3 oral)^b^	≤1	>1	≤2	>2
Ampicillin (2 g × 3 IV)	NT	NT	≤1	>1
Ampicillin (2 g × 4 IV)	NT	NT	≤1	>1
Azithromycin	≤0.25	>0.5	—	—
Cefaclor	≤0.001	>0.5	—	—
Cefdinir	—	—	—	—
Cefixime	—	—	≤0.12	>0.12
Cefotaxime	≤0.5	>2	≤0.12	>0.12
Cefpodoxime	≤0.25	>0.5	≤0.25	>0.25
Ceftibuten	—	—	≤1	>1
Ceftriaxone (1 g × 1 IV)	≤0.5	>2	≤0.12	>0.12
Ceftriaxone (2 g × 2 IV)	≤2	>2	≤0.12	>0.12
Cefuroxime^c^	≤0.25	>0.5	≤0.001	>1
Clarithromycin (0.25 g × 2 oral)	≤0.25	>0.5	—	—
Clarithromycin (0.5 g × 2 oral)	≤0.5	>0.5	—	—
Doxycycline	≤1	>2	NT	NT
Erythromycin (0.5 g × 2–4 oral or 0.5 g × 2–4 IV)	≤0.25	>0.5	NT	NT
Erythromycin (1 g × 4 oral or 1 g × 4 IV)	≤0.5	>0.5	NT	NT
Levofloxacin (0.5 g × 2 oral or 0.4 g × 2 IV)	≤0.001	>2	≤0.06	>0.06
Levofloxacin (0.75 g × 2 oral or 0.4 g × 3 IV)	≤2	>2	≤0.06	>0.06
Moxifloxacin	≤0.5	>0.5	≤0.12	>0.12
Penicillin (0.6 g 1 MU × 4 IV)	≤0.06	>2	NT	NT
Penicillin (2.4 g 2 MU × 4–6 IV)	≤2	>2	NT	NT
Tetracycline	≤1	>2	≤2	>2
Trimethoprim/sulfamethoxazole (0.16 g/0.8 g × 2 oral or IV)^d^	≤1	>2	≤0.5	>1
Trimethoprim/sulfamethoxazole (0.24 g/1.2 g × 2 oral or IV)^d^	≤2	>2	≤1	>1

—, not applicable; NT, not tested; R, resistant; S, susceptible.

^a^Where available, susceptibility was assessed using EUCAST higher dosage breakpoints.

^b^Amoxicillin/clavulanic acid was tested at a fixed concentration of 2 mg/L; breakpoints are expressed as the amoxicillin component.

^c^Breakpoints used are for cefuroxime axetil (oral).

^d^Trimethoprim/sulfamethoxazole was tested at a 1:19 trimethoprim to sulfamethoxazole ratio; breakpoints are expressed as the trimethoprim component.

**Table 3.** PK/PD MIC breakpoints (mg/L) used for *S. pneumoniae* and *H. influenzae* isolates

	*S. pneumoniae* and *H. influenzae*
Antimicrobial	S only
Amoxicillin (1.5 g/day)^a^	≤2
Amoxicillin (4 g/day)^b^	≤4
Amoxicillin/clavulanic acid^a^ (1.75 g/0.25 g/day adults; 45 mg/6.4 mg/kg/day children)	≤2
Amoxicillin/clavulanic acid^b^ (4 g/0.25 g/day adults; 90 mg/6.4 mg/kg/day children)	≤4
Ampicillin	—
Penicillin	—
Cefaclor	≤0.5
Cefdinir	≤0.25
Cefditoren	—
Cefixime	≤1
Cefpodoxime	≤0.5
Ceftriaxone	≤1
Cefuroxime^c^	≤1
Azithromycin	≤0.12
Clarithromycin	≤0.25
Erythromycin	≤0.25
Levofloxacin	≤2
Moxifloxacin	≤1
Trimethoprim/sulfamethoxazole^d^	≤0.5

—, not applicable; PK/PD, pharmacokinetic/pharmacodynamic; S, susceptible.

^a^Amoxicillin/clavulanic acid for low dose in adults/children.

^b^Amoxicillin/clavulanic acid for high dose in adults/children.

^c^Breakpoints used are for cefuroxime axetil (oral).

^d^Trimethoprim/sulfamethoxazole was tested at a 1:19 trimethoprim to sulfamethoxazole ratio; breakpoints are expressed as the trimethoprim component.

### Quality control and data analysis

Quality control strains *S. pneumoniae* ATCC 49619, *H. influenzae* ATCC 49247, *H. influenzae* ATCC 49766 and *E. coli* ATCC 32518 were included on each day of testing. The results of susceptibility testing were only accepted if the results of the quality control strains were within the published acceptable range. Differences in susceptibility (using CLSI criteria) across penicillin-susceptible isolates (*S. pneumoniae* only) were assessed for statistical significance with Fisher's exact test using XLSTAT version 2023.1.1.1399. A *P* value <0.05 was considered statistically significant. A similar statistical analysis was performed to compare the susceptibility of isolates from 2018 to 2021 with Pakistan SOAR data from 2015 to 2017 (using CLSI criteria).^17^

## Results

### *S. pneumoniae* isolates

A total of 57 *S. pneumoniae* isolates were collected from the Shaukat Khanum Memorial Cancer Hospital between 2018 and 2021. Most isolates came from sputum (*n* = 21, 36.8%), blood (*n* = 19, 33.3%), bronchoalveolar lavage (*n* = 3, 5.3%) and endotracheal aspirate (*n* = 2, 3.5%). The remaining isolates were from unidentified specimens (*n* = 12, 21.1%). The majority of isolates (*n* = 39, 68.4%) came from adolescent and adult patients (aged 13–64 years), and 10 (17.5%) isolates were from elderly patients (aged ≥65 years) and 8 (14.0%) isolates from paediatric patients (aged ≤12 years).

**Figure 1.**
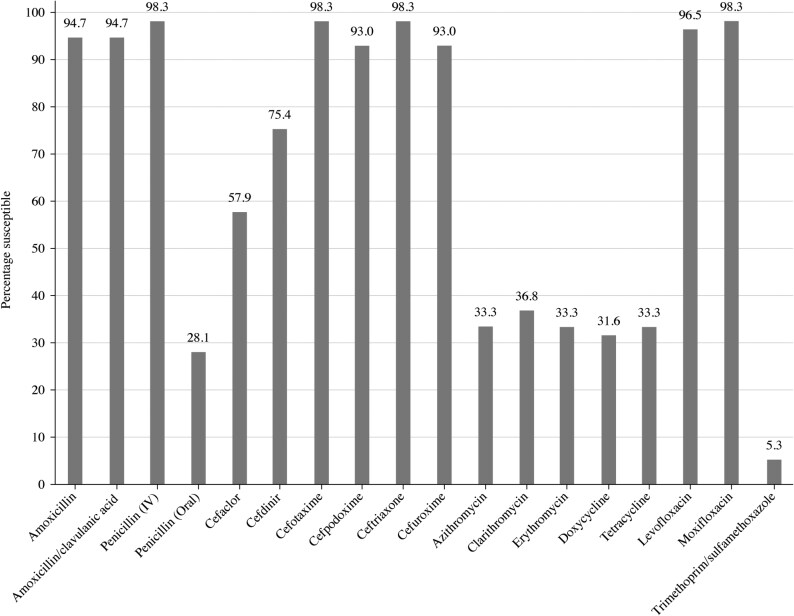
Antibiotic susceptibility rates of *S. pneumoniae* isolates (*n* = 57) from Pakistan based on CLSI breakpoints.

**Figure 2.**
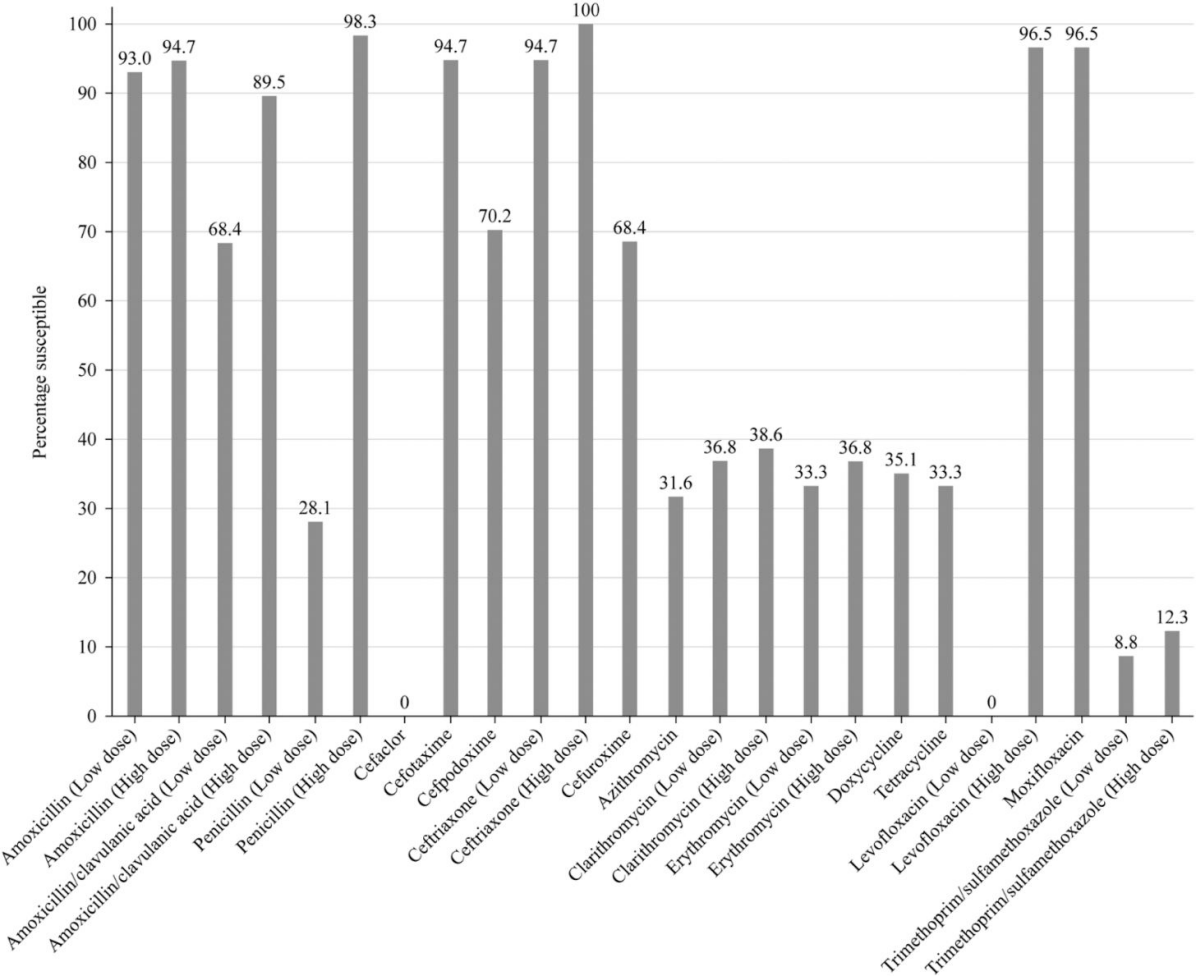
Antibiotic susceptibility rates of *S. pneumoniae* isolates (*n* = 57) from Pakistan based on EUCAST (dose-specific) breakpoints.

Summary MIC, susceptibility, and MIC distribution data for all *S. pneumoniae* isolates are given in Tables 4–6 and S1 (available as Supplementary data at *JAC* Online) and shown in Figures 1 and 2.

**Table 4.** MIC and susceptibility data for *S. pneumoniae* isolates (*n* = 57) from Pakistan using CLSI breakpoints

	MIC (mg/L)			CLSI susceptibility		
Antimicrobial	Range	50%	90%	%S	%I	%R
Amoxicillin	≤0.008–4	0.12	0.5	94.7	5.3	0
Amoxicillin/clavulanic acid (2:1)	≤0.008–8	0.12	0.5	94.7	3.5	1.8
Penicillin (2.4 g 2 MU × 4–6 IV)	≤0.008–4	0.12	0.5	98.2	1.8	0
Penicillin (oral)	≤0.008–4	0.12	0.5	28.1	66.7	5.3
Cefaclor	0.06–>4	1	>4	57.9	14.0	28.1
Cefdinir	0.03–8	0.25	1	75.4	17.5	7.0
Cefixime	≤0.25–>16	2	4	—	—	—
Cefotaxime	≤0.008–2	0.12	0.5	98.2	1.8	0
Cefpodoxime	≤0.015–4	0.25	0.5	93.0	1.8	5.3
Ceftibuten	1–>16	16	>16	—	—	—
Ceftriaxone	0.015–2	0.25	0.5	98.2	1.8	0
Cefuroxime	0.015–8	0.25	0.5	93.0	1.8	5.3
Azithromycin	≤0.015–>16	2	>16	33.3	5.3	61.4
Clarithromycin	≤0.015–>16	1	>16	36.8	1.8	61.4
Erythromycin	≤0.015–>16	2	>16	33.3	3.5	63.2
Doxycycline	0.03–>4	4	>4	31.6	0	68.4
Tetracycline	≤0.03–>4	>4	>4	33.3	0	66.7
Levofloxacin	≤0.12–>8	1	1	96.5	0	3.5
Moxifloxacin	≤0.03–2	0.06	0.12	98.2	1.8	0
Trimethoprim/sulfamethoxazole	≤0.06–>8	4	8	5.3	7.0	87.7

—, not applicable; I, intermediate; R, resistant; S, susceptible.

**Table 5.** MIC and susceptibility data for *S. pneumoniae* isolates (*n* = 57) from Pakistan using EUCAST (dose-specific) breakpoints

	MIC (mg/L)			EUCAST susceptibility		
Antimicrobial	Range	50%	90%	%S	%I	%R
Amoxicillin (0.5 g × 3 oral)	≤0.008–4	0.12	0.5	93.0	1.8	5.3
Amoxicillin (0.75–1 g × 3 oral)	≤0.008–4	0.12	0.5	94.7	—	5.3
Amoxicillin/clavulanic acid (0.5 g/0.125 g × 3 oral)	≤0.008–>8	0.5	2	68.4	21.1	10.5
Amoxicillin/clavulanic acid (0.875 g/0.125 g × 3 oral)	≤0.008–>8	0.5	2	89.5	—	10.5
Penicillin (0.6 g 1 MU × 4 IV)	≤0.008–4	0.12	0.5	28.1	70.2	1.8
Penicillin (2.4 g 2 MU × 4–6 IV)	≤0.008–4	0.12	0.5	98.3	—	1.8
Cefaclor	0.06–>4	1	>4	0	19.3	80.7
Cefdinir	0.03–8	0.25	1	—	—	—
Cefixime	≤0.25–>16	2	4	—	—	—
Cefotaxime	≤0.008–2	0.12	0.5	94.7	5.3	0
Cefpodoxime	≤0.015–4	0.25	0.5	70.2	22.8	7.0
Ceftibuten	1–>16	16	>16	—	—	—
Ceftriaxone (1 g × 1 IV)	0.015–2	0.25	0.5	94.7	5.3	0
Ceftriaxone (2 g × 2 IV)	0.015–2	0.25	0.5	100	—	0
Cefuroxime	0.015–8	0.25	0.5	68.4	22.8	8.8
Azithromycin	≤0.015–>16	2	>16	31.6	1.8	66.7
Clarithromycin (0.25 g × 2 oral)	≤0.015–>16	1	>16	36.8	1.8	61.4
Clarithromycin (0.5 g × 2 oral)	≤0.015–>16	1	>16	38.6	—	61.4
Erythromycin (0.5 g × 2–4 oral or 0.5 g × 2–4 IV)	≤0.015–>16	2	>16	33.3	3.5	63.2
Erythromycin (1 g × 4 oral or 1 g × 4 IV)	≤0.015–>16	2	>16	36.8	—	63.2
Doxycycline	0.03–>4	4	>4	35.1	10.5	54.4
Tetracycline	≤0.03–>4	>4	>4	33.3	0	66.7
Levofloxacin (0.5 g × 2 oral or 0.4 g × 2 IV)	≤0.12–>8	1	1	0	96.5	3.5
Levofloxacin (0.75 g × 2 oral or 0.4 g × 3 IV)	≤0.12–>8	1	1	96.5	—	3.5
Moxifloxacin	≤0.03–2	0.06	0.12	96.5	—	3.5
Trimethoprim/sulfamethoxazole (0.16 g/0.8 g × 2 oral or IV)	≤0.06–>8	4	8	8.8	3.5	87.7
Trimethoprim/sulfamethoxazole (0.24 g/1.2 g × 2 oral or IV)	≤0.06–>8	4	8	12.3	—	87.7

—, not applicable; I, susceptible, increased exposure; R, resistant; S, susceptible.

**Table 6.** Summary MIC and susceptibility data for *S. pneumoniae* (*n* = 57) from Pakistan using PK/PD breakpoints

	MIC (mg/L)			PK/PD susceptibility
Antimicrobial	Range	50%	90%	%S
Amoxicillin (1.5 g/day)	≤0.008–4	0.12	0.5	94.7
Amoxicillin (4 g/day)	≤0.008–4	0.12	0.5	100
Amoxicillin/clavulanic acid (1.75 g/0.25 g/day adults; 45 mg/6.4 mg/kg/day children)	≤0.008–8	0.12	0.5	94.7
Amoxicillin/clavulanic acid (4 g/0.25 g/day adults; 90 mg/6.4 mg/kg/day children)	≤0.008–8	0.12	0.5	98.2
Penicillin	≤0.008–4	0.12	0.5	—
Cefaclor	0.06–>4	1	>4	19.3
Cefdinir	0.03–8	0.25	1	61.4
Cefixime	≤0.25–>16	2	4	45.6
Cefotaxime	≤0.008–2	0.12	0.5	—
Cefpodoxime	≤0.015–4	0.25	0.5	93.0
Ceftibuten	1–>16	16	>16	—
Ceftriaxone	0.015–2	0.25	0.5	98.2
Cefuroxime	0.015–8	0.25	0.5	93.0
Azithromycin	≤0.015–>16	2	>16	31.6
Clarithromycin	≤0.015–>16	1	>16	36.8
Erythromycin	≤0.015–>16	2	>16	33.3
Doxycycline	0.03–>4	4	>4	31.6
Tetracycline	≤0.03–>4	>4	>4	—
Levofloxacin	≤0.12–>8	1	1	96.5
Moxifloxacin	≤0.03–2	0.06	0.12	98.2
Trimethoprim/sulfamethoxazole	≤0.06–>8	4	8	5.3

—, not applicable; PK/PD, pharmacokinetic/pharmacodynamic; S, susceptible.

### *S. pneumoniae* susceptibility

The proportion of penicillin-susceptible pneumococci from Pakistan following CLSI oral or EUCAST low-dose IV breakpoints was 28.1% but increased to 98.3% and 98.2% with EUCAST high-dose and CLSI IV breakpoints, respectively. The rates of penicillin-intermediate and penicillin-resistant pneumococci using CLSI oral or EUCAST low-dose breakpoints were 66.7% and 5.3%, respectively. Following CLSI and PK/PD breakpoints, amoxicillin, amoxicillin/clavulanic acid, second-generation cephalosporins (cefpodoxime and cefuroxime) and third-generation cephalosporins (ceftriaxone and cefotaxime) showed similar activity with susceptibility ≥93.0%, but the second-generation and third-generation cephalosporins cefaclor and cefdinir were less active (57.9% and 75.4% susceptible, respectively, by CLSI and 19.3% and 61.4% susceptible, respectively, by PK/PD breakpoints). The use of EUCAST breakpoints produced similar susceptibility (≥89.5%) for amoxicillin and amoxicillin/clavulanic acid (high dose only), cefotaxime and ceftriaxone, but susceptibility to cefaclor (0%), cefpodoxime (70.2%) and cefuroxime (68.4%) was lower than that obtained with CLSI or PK/PD breakpoints. Poor activity (5.3%–38.6% susceptibility) was observed for the macrolides (azithromycin, clarithromycin and erythromycin), tetracyclines (doxycycline and tetracycline) and trimethoprim/sulfamethoxazole by CLSI, EUCAST and PK/PD interpretation. Moxifloxacin susceptibility was 96.5% by EUCAST and 98.2% by CLSI and PK/PD, and levofloxacin susceptibility was 96.5% by CLSI, PK/PD and EUCAST high dose (Tables 4–6 and Figures 1 and 2).

### Comparative susceptibility of *S. pneumoniae* collected in 2015–17 and 2018–21

**Figure 3.**
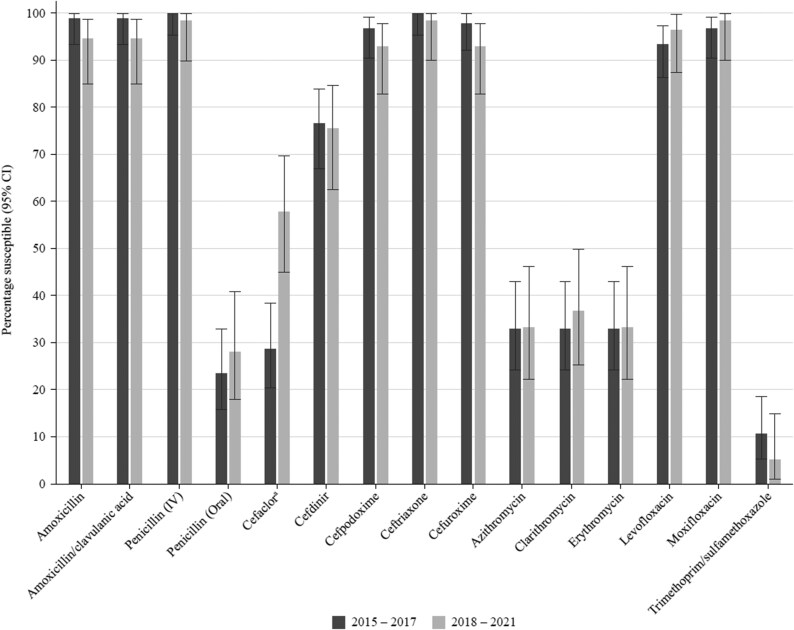
Comparison of antibiotic susceptibility rates of *S. pneumoniae* isolates from Pakistan collected in 2015–17 with isolates collected in 2018–21 (CLSI breakpoints). ^a^Susceptibility was significantly higher in 2018–21 than in 2015–17 (*P* = 0.0006).

Data for the period 2015–17 have previously been published from the SOAR surveillance study and were compared for mutually tested antibiotics with the current study (2018–21) (Figure 3). There was no significant change in susceptibility except for an increased level of susceptibility to cefaclor (26.7% versus 57.9%). However, this improved activity would still be considered inadequate for therapeutic consideration.

### *H. influenzae* isolates

A total of 67 *H. influenzae* isolates were collected from the Shaukat Khanum Memorial Cancer Hospital between 2018 and 2021. Most isolates originated from sputum (*n* = 44, 65.7%). The remaining isolates were from bronchoalveolar lavage (*n* = 4, 6.0%), endotracheal aspirate (*n* = 3, 4.5%), blood (*n* = 1, 1.5%), middle ear (*n* = 1, 1.5%) and unidentified specimens (*n* = 14, 20.9%). Most isolates (*n* = 44, 65.7%) came from adolescent and adult patients (aged 13–64 years), 17 (25.4%) isolates were from paediatric patients (aged ≤12 years), and six (9.0%) isolates were from elderly patients (aged ≥65 years).

**Figure 4.**
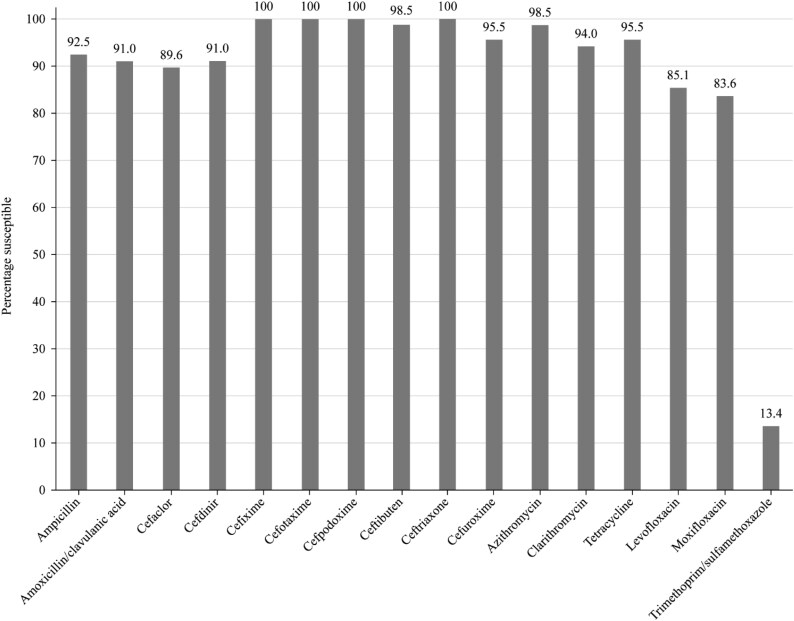
Antibiotic susceptibility rates of *H. influenzae* isolates (*n* = 67) from Pakistan based on CLSI breakpoints.

**Figure 5.**
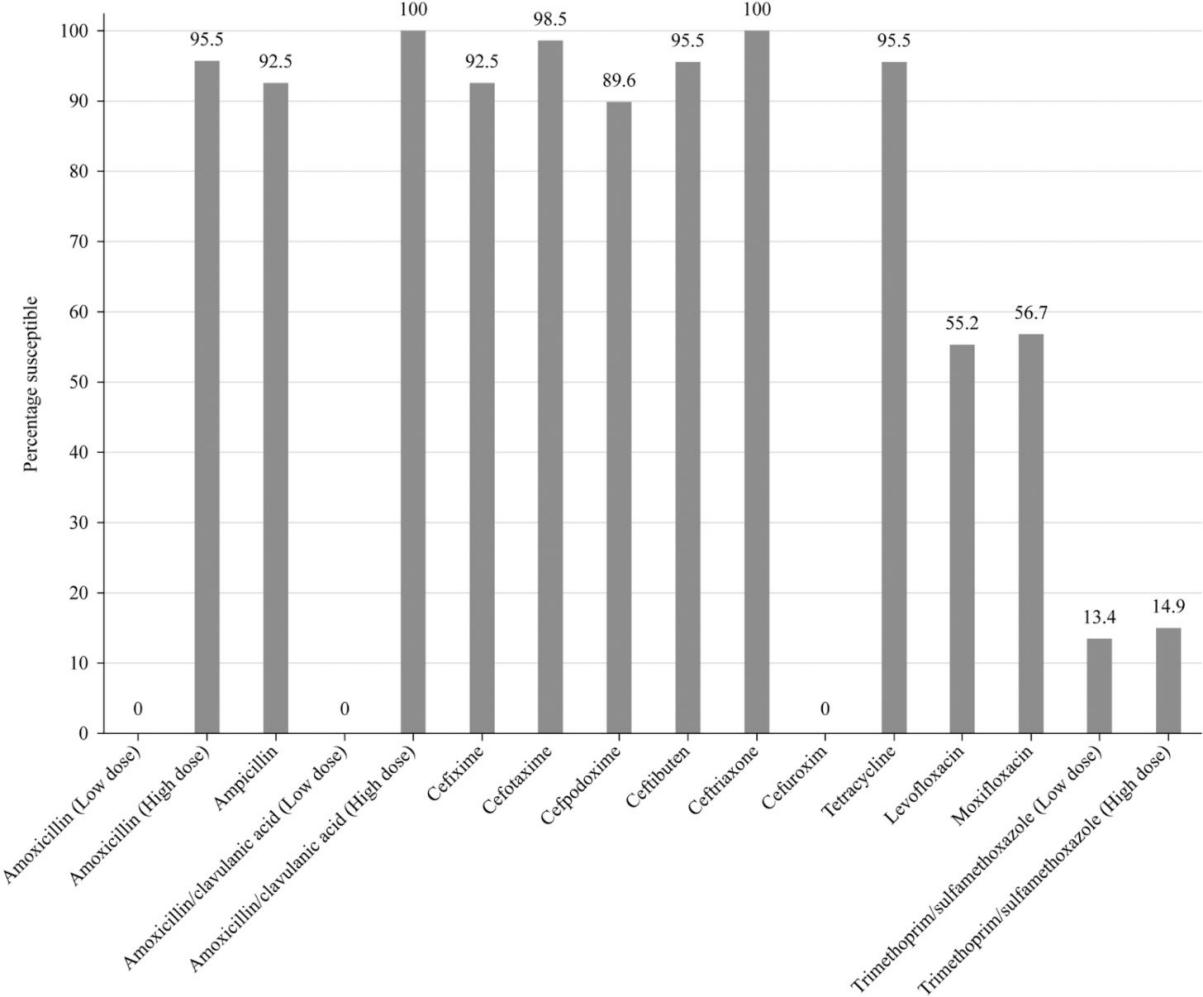
Antibiotic susceptibility rates of *H. influenzae* isolates (*n* = 67) from Pakistan based on EUCAST (dose-specific) breakpoints.

Summary MIC, susceptibility and MIC distribution data for all 67 *H. influenzae* isolates are shown in Tables 7–9 and S2 and Figures 4 and 5.

**Table 7.** MIC and susceptibility data for *H. influenzae* isolates (*n* = 67) from Pakistan using CLSI breakpoints

	MIC (mg/L)			CLSI susceptibility		
Antimicrobial	Range	50%	90%	%S	%I	%R
Amoxicillin	≤0.03–128	0.5	2	—	—	—
Ampicillin	≤0.03–64	0.25	1	92.5	3.0	4.5
Amoxicillin/clavulanic acid (2:1)	0.12–4	0.5	2	91.1	9.0	0
Cefaclor	≤0.25–32	2	16	89.6	6.0	4.5
Cefdinir	≤0.06–2	0.25	1	91.0	—	—
Cefixime	≤0.008–1	0.03	0.12	100	—	—
Cefotaxime	≤0.002–0.25	0.008	0.12	100	—	—
Cefpodoxime	≤0.015–2	0.06	0.5	100	—	—
Ceftibuten	0.015–4	0.06	1	98.5	—	—
Ceftriaxone	≤0.001–0.06	0.004	0.03	100	—	—
Cefuroxime	0.06–16	0.5	4	95.5	1.5	3.0
Azithromycin	≤0.12–>8	0.5	2	98.5	—	—
Clarithromycin	≤0.25–>32	4	8	94.0	4.5	1.5
Tetracycline	≤0.12–16	0.25	0.5	95.5	0	4.5
Levofloxacin	0.008–>8	0.06	>8	85.1	—	—
Moxifloxacin	0.008–>8	0.06	8	83.6	—	—
Trimethoprim/sulfamethoxazole	≤0.008–>8	8	>8	13.4	1.5	85.1

—, not applicable; I, intermediate; R, resistant; S, susceptible.

**Table 8.** MIC and susceptibility data for *H. influenzae* isolates (*n* = 67) from Pakistan using EUCAST (dose-specific) breakpoints

	MIC (mg/L)			EUCAST susceptibility		
Antimicrobial	Range	50%	90%	%S	%I	%R
Amoxicillin (0.5 g × 3 oral)	≤0.03–128	0.5	2	0	95.5	4.5
Amoxicillin (0.75–1 g × 3 oral)	≤0.03–128	0.5	2	95.5	—	4.5
Ampicillin	≤0.03–64	0.25	1	92.5	—	7.5
Amoxicillin/clavulanic acid (0.5 g/0.125 g × 3 oral)	≤0.03–2	0.25	2	0	100	0
Amoxicillin/clavulanic acid (0.875 g/0.125 g × 3 oral)	≤0.03–2	0.25	2	100	—	0
Cefaclor	≤0.25–32	2	16	—	—	—
Cefdinir	≤0.06–2	0.25	1	—	—	—
Cefixime	≤0.008–1	0.03	0.12	92.5	—	7.5
Cefotaxime	≤0.002–0.25	0.008	0.12	98.5	—	1.5
Cefpodoxime	≤0.015–2	0.06	0.5	89.6	—	10.4
Ceftibuten	0.015–4	0.06	1	95.5	—	4.5
Ceftriaxone	≤0.001–0.06	0.004	0.03	100	—	0
Cefuroxime	0.06–16	0.5	4	0	67.2	32.8
Azithromycin	≤0.12–>8	0.5	2	—	—	—
Clarithromycin	≤0.25–>32	4	8	—	—	—
Tetracycline	≤0.12–16	0.25	0.5	95.5	—	4.5
Levofloxacin	0.008–>8	0.06	>8	55.2	—	44.8
Moxifloxacin	0.008–>8	0.06	8	56.7	—	43.3
Trimethoprim/sulfamethoxazole (0.16 g/0.8 g × 2 oral or IV)	≤0.008–>8	8	>8	13.4	1.5	85.1
Trimethoprim/sulfamethoxazole (0.24 g/1.2 g × 2 oral or IV)	≤0.008–>8	8	>8	14.9	—	85.1

—, not applicable; I, susceptible, increased exposure; R, resistant; S, susceptible.

**Table 9.** Summary MIC and susceptibility data for *H. influenzae* (*n* = 67) from Pakistan using PK/PD breakpoints

	MIC (mg/L)			PK/PD susceptibility
Antimicrobial	Range	50%	90%	%S
Amoxicillin (1.5 g/day)	≤0.03–128	0.5	2	95.5
Amoxicillin (4 g/day)	≤0.03–128	0.5	2	95.5
Amoxicillin/clavulanic acid (1.75 g/0.25 g/day adults; 45 mg/6.4 mg/kg/day children)	≤0.03–2	0.25	2	95.5
Amoxicillin/clavulanic acid (4 g/0.25 g/day adults; 90 mg/6.4 mg/kg/day children)	≤0.03–2	0.25	2	97.0
Ampicillin	≤0.03–64	0.25	1	—
Cefaclor	≤0.25–32	2	16	17.9
Cefdinir	≤0.06–2	0.25	1	68.7
Cefixime	≤0.008–1	0.03	0.12	100
Cefotaxime	≤0.002–0.25	0.008	0.12	—
Cefpodoxime	≤0.015–2	0.06	0.5	95.5
Ceftibuten	0.015–4	0.06	1	—
Ceftriaxone	≤0.001–0.06	0.004	0.03	100
Cefuroxime	0.06–16	0.5	4	67.2
Azithromycin	≤0.12–>8	0.5	2	4.5
Clarithromycin	≤0.25–>32	4	8	3.0
Tetracycline	≤0.12–16	0.25	0.5	—
Levofloxacin	0.008–>8	0.06	>8	85.1
Moxifloxacin	0.008–>8	0.06	8	83.6
Trimethoprim/sulfamethoxazole	≤0.008–>8	8	>8	13.4

—, not applicable; PK/PD, pharmacokinetic/pharmacodynamic; S, susceptible.

### *H. influenzae* susceptibility

Virtually all isolates of *H. influenzae* were β-lactamase negative (64/67, 95.5%). Within this population, two isolates were β-lactamase negative ampicillin-resistant (BLNAR) by EUCAST breakpoints (ampicillin MIC ≥2 mg/L) and none by CLSI breakpoints (ampicillin MIC ≥4 mg/L). Isolates from Pakistan test antibiotics were ≥83.6% susceptible to all antibiotics according to CLSI and PK/PD breakpoints, except for cefaclor (17.9% by PK/PD), cefdinir (68.7% by PK/PD), cefuroxime (67.2% by PK/PD), macrolides (azithromycin 4.5% and clarithromycin 3.0% by PK/PD) and trimethoprim/sulfamethoxazole (13.4% susceptible by CLSI and PK/PD). Similar results were obtained when using EUCAST breakpoints, provided high-dose regimens were used for amoxicillin and amoxicillin/clavulanate. Susceptibility rates of 0% and 55.2%/56.7% were found for cefuroxime and fluoroquinolone (levofloxacin/moxifloxacin), respectively, using EUCAST breakpoints (Tables 7–9 and Figures 4 and 5). Macrolide breakpoints are not provided by EUCAST against *H. influenzae.*

### Comparative susceptibility of *H. influenzae* collected in 2015–17 and 2018–21

**Figure 6.**
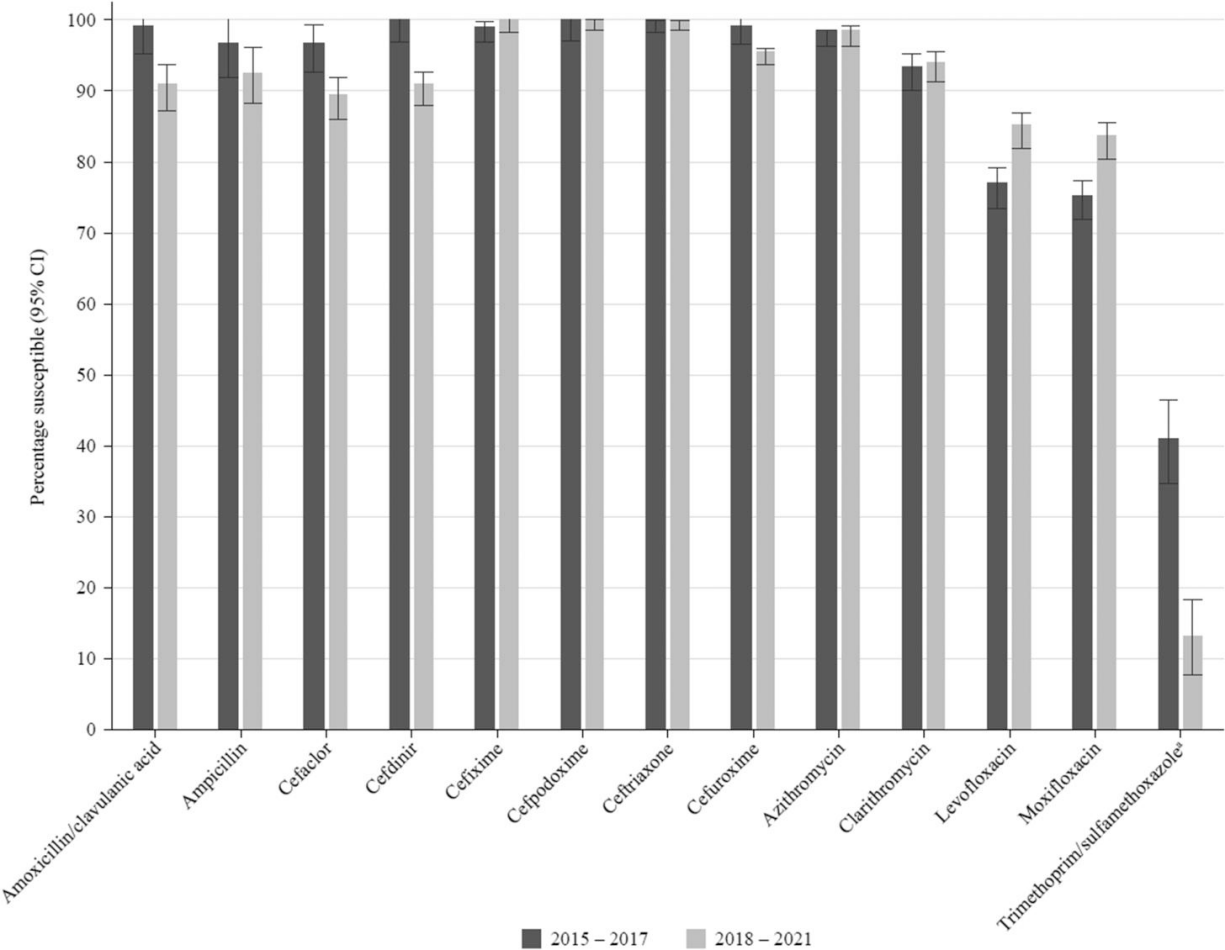
Comparison of antibiotic susceptibility rates of *H. influenzae* isolates from Pakistan collected in 2015–17 with isolates collected in 2018–21 (CLSI breakpoints). ^a^Susceptibility was significantly lower in 2018–21 than in 2015–17 (*P* < 0.0001).

Generally, there was no significant change in susceptibility when comparing data from 2015 to 2017 with 2018–21 using CLSI breakpoints, with the exception of trimethoprim/sulfamethoxazole, where susceptibility significantly reduced since 2015–17 (Figure 6).

## Discussion

SOAR is an ongoing global surveillance study focusing on the two main CA-RTIs pathogens, *S. pneumoniae* and *H. influenzae*, that has monitored numerous countries since 2002, including Pakistan. The data presented here are an analysis of the antibiotic susceptibility of *S. pneumoniae* and *H. influenzae* isolates collected from the Shaukat Khanum Memorial Cancer Hospital, Lahore, Pakistan, between 2018 and 2021. A potential limitation of this study is the national antibiotic resistance representation as the samples were collected from only one centre; however, as most isolates were from community-acquired infections and presumably unrelated, this is likely representative of the broader Pakistani community. This centre was one of two that participated in the previous SOAR surveillance from 2015 to 2017,^17^ and a direct statistical comparison between the two study periods is presented here.

The penicillin susceptibility results for *S. pneumoniae* in Pakistan from 2018 to 2021 confirm that oral penicillin or low-dose IV penicillin is not an appropriate therapy for CA-RTIs, as only 28.1% of pneumococci were susceptible using EUCAST low-dose IV or CLSI oral breakpoints. Although this shows a numerical increase compared with SOAR 2015–17 data (23.4% susceptible), the increase was not statistically significant.^17^ It is possible that susceptibility to oral penicillin reached a plateau after the decline since SOAR 2002–03, when oral penicillin susceptibility in Pakistan was 90.0%.^18,19^ Both CLSI and EUCAST guidelines, on the other hand, indicate that higher-dose IV penicillin is a superior option, with susceptibility at 98.3%. CLSI and PK/PD breakpoints indicate a comparably high level of susceptibility to amoxicillin, amoxicillin/clavulanic acid and cephalosporins (excluding cefaclor and cefdinir). Susceptibility using EUCAST breakpoints for most β-lactams was also in keeping with CLSI if high-dose amoxicillin/clavulanic acid was used. However, cefaclor, cefpodoxime and cefuroxime susceptibility was lower following EUCAST guidelines. Susceptibility according to either guideline indicated poor activity for macrolides, tetracyclines and trimethoprim/sulfamethoxazole, but good activity for fluoroquinolones against *S. pneumoniae* from Pakistan. Outside of the SOAR study, published antimicrobial susceptibility data for CA-RTI isolates from Pakistan are rare, but one recently published longitudinal study from 1993 to 2016 at a tertiary hospital in Karachi confirmed high resistance to trimethoprim/sulfamethoxazole over the whole period, and a trend of increased resistance to tetracycline and erythromycin, with low fluoroquinolone resistance in pneumococci.^20^ It is interesting that although the authors found no change in penicillin resistance for non- CSF isolates, there was a marked increase in penicillin resistance for CSF isolates of *S. pneumoniae* since 2008.

We compared the susceptibility of pneumococci using CLSI breakpoints for isolates previously collected in 2015–17 from Pakistan with susceptibility from the current study (2018–21). As noted above for oral penicillin, there was no significant difference in susceptibility between the two study periods, except for cefaclor, where susceptibility increased. Although this increase was statistically significant (*P* = 0.0006), the per cent susceptibility remained low.

All but three *H. influenzae* isolates from Pakistan were β-lactamase negative (95.5%), with two being BLNAR according to EUCAST breakpoints. Susceptibility to antibiotics was ≥83.6% by CLSI breakpoints, except for trimethoprim/sulfamethoxazole (13.4% susceptible). SOAR surveillance from 2002 to 2015 also indicated generally high antibiotic susceptibility with *H. influenzae*, except trimethoprim/sulfamethoxazole and chloramphenicol; the latter antibiotic was not tested in the current study.^18^ The only statistically significant difference between 2015–17 and 2018–21 susceptibility by CLSI breakpoints was a reduction in trimethoprim/sulfamethoxazole susceptibility. However, there were differences in susceptibility between CLSI and EUCAST in 2019–21 for cefuroxime (0% EUCAST susceptible versus 95.5% CLSI susceptible), macrolides (due to no EUCAST breakpoints given), levofloxacin (85.1% susceptible by CLSI versus 55.2% by EUCAST) and moxifloxacin (83.6% versus 56.7%). The lower fluoroquinolone susceptibility by EUCAST compared with CLSI was also observed in the previous SOAR surveillance for 2015–17 in Pakistan: levofloxacin (33.6% versus 77.1% susceptible) and moxifloxacin (34.4% versus 75.4% susceptible).^17^ Data from the ATLAS surveillance interactive database for *H. influenzae* collected from 2018 to 2021 and originating from 57 countries (3896 isolates in total) showed that levofloxacin susceptibility by EUCAST breakpoints was 95.2% overall.^21^ Therefore, the large difference in fluoroquinolone susceptibility between CLSI and EUCAST is unexpected. The ATLAS database for 2018–21 does not include Pakistan, but isolates from India were only 25% levofloxacin-susceptible by EUCAST breakpoints.^21^ The levofloxacin-resistant isolates by EUCAST breakpoints are so-called non-wild type, but most would still be considered susceptible by CLSI. It is possible, therefore, that dominant levofloxacin-non-wild-type (but levofloxacin-susceptible by CLSI breakpoints) isolates have been circulating in Asia since 2015.

In summary, although some resistance was observed, there are many suitable therapeutic options for the treatment of *S. pneumoniae* and *H. influenzae* originating from CA-RTIs in Pakistan despite some concerns about high levels of inappropriate antibiotic use in this country. Continued surveillance of antibiotic susceptibility in Pakistan is required to regularly assess any future changes.

## Supplementary Material

dkaf288_Supplementary_Data

## Supplementary data

Tables S1 and S2 are available as Supplementary data at *JAC* Online.

## References

[1] Aliberti S, Dela Cruz CS, Amati F et al Community-acquired pneumonia. Lancet 2021; 398: 906–19. 10.1016/S0140-6736(21)00630-9 34481570

[2] Cillóniz C, Dominedò C, Garcia-Vidal C et al Community-acquired pneumonia as an emergency condition. Curr Opin Crit Care 2018; 24: 531–9. 10.1097/MCC.0000000000000550 30239410

[3] Saleem Z, Hassali MA, Godman B et al Sale of WHO AWaRe groups antibiotics without a prescription in Pakistan: a simulated client study. J Pharm Policy Pract 2020; 13: 26. 10.1186/s40545-020-00233-3 32774870 PMC7397594

[4] Saleem Z, Faller EM, Godman B et al Antibiotic consumption at community pharmacies: a multicenter repeated prevalence surveillance using WHO methodology. Med Access Point Care 2021; 5: 1–9. 10.1177/23992026211064714 PMC9413637 36204499

[5] Dhedhi NA, Ashraf H, Ansari NB et al Self-medication among people visiting outpatient clinics of a tertiary care hospital, Karachi. J Family Med Prim Care 2021; 10: 773–9. 10.4103/jfmpc.jfmpc_1887_20 34041075 PMC8138398

[6] King LM, Fleming-Dutra KE, Hicks LA. Advances in optimizing the prescription of antibiotics in outpatient settings. BMJ 2018; 363; k3047. 10.1136/bmj.k3047 30420401 PMC6511972

[7] Jain S, Self WH, Wunderink RG et al Community-acquired pneumonia requiring hospitalization among U.S. adults. N Engl J Med 2015; 373: 415–27. 10.1056/NEJMoa1500245 26172429 PMC4728150

[8] Gadsby NJ, Russell CD, McHugh MP et al Comprehensive molecular testing for respiratory pathogens in community-acquired pneumonia. Clin Infect Dis 2016; 62: 817–23. 10.1093/cid/civ1214 26747825 PMC4787606

[9] Peyrani P, Mandell L, Torres A et al The burden of community-acquired bacterial pneumonia in the era of antibiotic resistance. Expert Rev Respir Med 2019; 13: 139–52. 10.1080/17476348.2019.1562339 30596308

[10] Heinz E . The return of Pfeiffer's bacillus: rising incidence of ampicillin resistance in *Haemophilus influenzae*. Microb Genom 2018; 4: e000214. 10.1099/mgen.0.000214 30207515 PMC6202453

[11] World Health Organization . *Global antimicrobial resistance and use surveillance system (GLASS)*. https://www.who.int/initiatives/glass.

[12] Torumkuney D, Chaiwarith R, Reechaipichitkul W et al Results from the Survey of Antibiotic Resistance (SOAR) 2012–14 in Thailand, India, South Korea and Singapore. J Antimicrob Chemother 2016; 71: 3628. 10.1093/jac/dkw332 27559118 PMC7297303

[13] CLSI . Methods for Dilution Antimicrobial Susceptibility Tests for Bacteria That Grow Aerobically―Twelfth Edition: M07. 2024 https://clsi.org/shop/standards/m07/.

[14] CLSI . Performance Standards for Antimicrobial Susceptibility Testing―Thirty-Fifth Edition: M100. 2025 https://clsi.org/shop/standards/m100/.

[15] European Committee on Antimicrobial Susceptibility Testing . Breakpoint tables for interpretation of MICs and zone diameters. Version 12.0, 2022. http://www.eucast.org/fileadmin/src/media/PDFs/EUCAST_files/Breakpoint_tables/v_12.0_Breakpoint_Tables.pdf.

[16] Anon JB, Jacobs MR, Poole MD et al Antimicrobial treatment guidelines for acute bacterial rhinosinusitis. Otolaryngol Head Neck Surg 2004; 130(Suppl 1): 1–45. 10.1016/j.otohns.2003.12.003 14726904 PMC7118847

[17] Torumkuney D, Anwar S, Nizamuddin S et al Results from the Survey of Antibiotic Resistance (SOAR) 2015-17 in Pakistan: data based on CLSI, EUCAST (dose-specific) and pharmacokinetic/pharmacodynamic (PK/PD) breakpoints. J Antimicrob Chemother 2020; 75(Suppl 1): i76–87. 10.1093/jac/dkaa085 32337594

[18] Zafar A, Hasan R, Nizamuddin S et al Antibiotic susceptibility in *Streptococcus pneumoniae*, *Haemophilus influenzae* and *Streptococcus pyogenes* in Pakistan: a review of results from the Survey of Antibiotic Resistance (SOAR) 2002-15. J Antimicrob Chemother 2016; 71(Suppl 1): i103–9. 10.1093/jac/dkw076 27048578 PMC4890355

[19] Torumkuney D, Jamil B, Nizamuddin S et al Country data on AMR in Pakistan in the context of community-acquired respiratory tract infections: links between antibiotic susceptibility, local and international antibiotic prescribing guidelines, access to medicine and clinical outcome. J Antimicrob Chemother 2022; 77(Suppl_1): i18–25. 10.1093/jac/dkac213 36065729 PMC9445852

[20] Zafar A, Lalani FK, Longi AA et al Increase in penicillin and multidrug resistance in *Streptococcus pneumoniae* (1993-2016): report from a tertiary care hospital laboratory, Pakistan. J Pak Med Assoc 2021; 71: 2726–30. 10.47391/JPMA.1178 35150528

[21] Pfizer . *Antimicrobial surveillance*. https://www.pfizer.com/science/therapeutic-areas/anti-infectives/antimicrobial-surveillance.

