# Supplementary material for: Results from the Survey of Antibiotic Resistance (SOAR) 2018–21 in Pakistan: data based on CLSI, EUCAST (dose-specific) and pharmacokinetic/pharmacodynamic (PK/PD) breakpoints
Source: J Antimicrob Chemother. 2025 Nov 24;80(Suppl 3):iii83–97. doi: 10.1093/jac/dkaf288 (PMC12641124; doi:10.1093/jac/dkaf288)
Supplement: dkaf288_Supplementary_Data [file dkaf288_supplementary_data.docx]

**Results from the Survey of Antibiotic Resistance (SOAR) 2018 – 21 in Pakistan: data based on CLSI, EUCAST (dose-specific) and pharmacokinetic/pharmacodynamic (PK/PD) breakpoints**

**Authors:** Didem TORUMKUNEY^1^, Summiya NIZAMUDDIN^2^, Ian MORRISSEY^3^, Rendani MANENZHE^4^, Anand MANOHARAN^5*^

**Affiliations:** ^1^GSK, London, UK; ^2^Shaukat Khanum Memorial Cancer Hospital and Research Centre, Lahore, Pakistan; ^3^Antimicrobial Focus Ltd., Sawbridgeworth, UK; ^4^GSK, Gauteng, South Africa; ^5^Infectious Diseases Medical & Scientific Affairs, GSK, Mumbai, India

*Corresponding author. E-mail: [anand.x.manoharan@gsk.com](mailto:anand.x.manoharan@gsk.com)

**Running title:** Survey of Antibiotic Resistance (SOAR) in Pakistan in 2018 – 21

**Supplementary Table 1.** MIC distribution data for *S. pneumoniae* isolates (*n* = 57) from Pakistan

|  |  | Number of isolates at MIC (mg/L) | | | | | | | | | | | | | | | | | | | | |
| --- | --- | --- | --- | --- | --- | --- | --- | --- | --- | --- | --- | --- | --- | --- | --- | --- | --- | --- | --- | --- | --- | --- |
| Antimicrobial |  | ≤0.008 | ≤0.015 | 0.015 | ≤0.03 | 0.03 | ≤0.06 | 0.06 | ≤0.12 | 0.12 | ≤0.25 | 0.25 | ≤0.5 | 0.5 | 1 | 2 | 4 | >4 | 8 | >8 | 16 | >16 |
| AMX | N | 3 | – | 2 | – | 6 | – | 3 | – | 18 | – | 17 | – | 4 | 1 | – | 3 | – | – | – | – | – |
|  | Cum. % | 5.3 | – | 8.8 | – | 19.3 | – | 24.6 | – | 56.1 | – | 86.0 | – | 93.0 | 94.7 | – | 100 | – | – | – | – | – |
|  | % | 5.3 | – | 3.5 | – | 10.5 | – | 5.3 | – | 31.6 | – | 29.8 | – | 7.0 | 1.8 | – | 5.3 | – | – | – | – | – |
| AMC (2:1) | N | 3 | – | 3 | – | 5 | – | 3 | – | 16 | – | 17 | – | 6 | 1 | – | 2 | – | 1 | – | – | – |
|  | Cum. % | 5.3 | – | 10.5 | – | 19.3 | – | 24.6 | – | 52.6 | – | 82.5 | – | 93.0 | 94.7 | – | 98.2 | – | 100 | – | – | – |
|  | % | 5.3 | – | 5.3 | – | 8.8 | – | 5.3 | – | 28.1 | – | 29.8 | – | 10.5 | 1.8 | – | 3.5 | – | 1.8 | – | – | – |
| AMC  [2 mg/L] | N | 1 | – | 2 | – | 3 | – | 5 | – | 3 | – | 10 | – | 15 | 12 | 2 | 1 | – | 1 | 2 | – | – |
|  | Cum. % | 1.8 | – | 5.3 | – | 10.5 | – | 19.3 | – | 24.6 | – | 42.1 | – | 68.4 | 89.5 | 93.0 | 94.7 | – | 96.5 | 100 | – | – |
|  | % | 1.8 | – | 3.5 | – | 5.3 | – | 8.8 | – | 5.3 | – | 17.5 | – | 26.3 | 21.1 | 3.5 | 1.8 | – | 1.8 | 3.5 | – | – |
| AZM | N | – | 1 | – | – | 3 | – | 12 | – | 2 | – | – | – | 1 | 3 | 7 | 12 | – | 1 | – | – | 15 |
|  | Cum. % | – | 1.8 | – | – | 7.0 | – | 28.1 | – | 31.6 | – | – | – | 33.3 | 38.6 | 50.9 | 71.9 | – | 73.7 | – | – | 100 |
|  | % | – | 1.8 | – | – | 5.3 | – | 21.1 | – | 3.5 | – | – | – | 1.8 | 5.3 | 12.3 | 21.1 | – | 1.8 | – | – | 26.3 |
| CEC | N | – | – | – | – | – | – | 1 | – | 1 | – | 3 | – | 6 | 22 | 8 | 8 | 8 | – | – | – | – |
|  | Cum. % | – | – | – | – | – | – | 1.8 | – | 3.5 | – | 8.8 | – | 19.3 | 57.9 | 71.9 | 86.0 | 100 | – | – | – | – |
|  | % | – | – | – | – | – | – | 1.8 | – | 1.8 | – | 5.3 | – | 10.5 | 38.6 | 14.0 | 14.0 | 14.0 | – | – | – | – |
| CDR | N | – | – | – | – | 1 | – | 8 | – | 4 | – | 22 | – | 8 | 10 | 1 | 2 | – | 1 | – | – | – |
|  | Cum. % | – | – | – | – | 1.8 | – | 15.8 | – | 22.8 | – | 61.4 | – | 75.4 | 93.0 | 94.7 | 98.2 | – | 100 | – | – | – |
|  | % | – | – | – | – | 1.8 | – | 14.0 | – | 7.0 | – | 38.6 | – | 14.0 | 17.5 | 1.8 | 3.5 | – | 1.8 | – | – | – |
| CFM | N | – | – | – | – | – | – | – | – | – | 7 | – | – | 6 | 13 | 14 | 12 | – | 2 | – | 2 | 1 |
|  | Cum. % | – | – | – | – | – | – | – | – | – | 12.3 | – | – | 22.8 | 45.6 | 70.2 | 91.2 | – | 94.7 | – | 98.2 | 100 |
|  | % | – | – | – | – | – | – | – | – | – | 12.3 | – | – | 10.5 | 22.8 | 24.6 | 21.1 | – | 3.5 | – | 3.5 | 1.8 |
| CTX | N | 1 | – | 5 | – | 3 | – | 2 | – | 25 | – | 15 | – | 3 | 2 | 1 | – | – | – | – | – | – |
|  | Cum. % | 1.8 | – | 10.5 | – | 15.8 | – | 19.3 | – | 63.2 | – | 89.5 | – | 94.7 | 98.2 | 100 | – | – | – | – | – | – |
|  | % | 1.8 | – | 8.8 | – | 5.3 | – | 3.5 | – | 43.9 | – | 26.3 | – | 5.3 | 3.5 | 1.8 | – | – | – | – | – | – |
| CPD | N | – | 1 | – | – | 7 | – | 2 | – | 9 | – | 21 | – | 13 | 1 | 2 | 1 | – | – | – | – | – |
|  | Cum. % | – | 1.8 | – | – | 14.0 | – | 17.5 | – | 33.3 | – | 70.2 | – | 93.0 | 94.7 | 98.2 | 100 | – | – | – | – | – |
|  | % | – | 1.8 | – | – | 12.3 | – | 3.5 | – | 15.8 | – | 36.8 | – | 22.8 | 1.8 | 3.5 | 1.8 | – | – | – | – | – |
| CTB | N | – | – | – | – | – | – | – | – | – | – | – | – | – | 1 | 3 | 6 | – | 15 | – | 12 | 20 |
|  | Cum. % | – | – | – | – | – | – | – | – | – | – | – | – | – | 1.8 | 7.0 | 17.5 | – | 43.9 | – | 64.9 | 100 |
|  | % | – | – | – | – | – | – | – | – | – | – | – | – | – | 1.8 | 5.3 | 10.5 | – | 26.3 | – | 21.1 | 35.1 |
| CRO | N | – | – | 4 | – | 4 | – | 2 | – | 11 | – | 27 | – | 6 | 2 | 1 | – | – | – | – | – | – |
|  | Cum. % | – | – | 7.0 | – | 14.0 | – | 17.5 | – | 36.8 | – | 84.2 | – | 94.7 | 98.2 | 100 | – | – | – | – | – | – |
|  | % | – | – | 7.0 | – | 7.0 | – | 3.5 | – | 19.3 | – | 47.4 | – | 10.5 | 3.5 | 1.8 | – | – | – | – | – | – |
| CXM | N | – | – | 5 | – | 2 | – | 1 | – | 17 | – | 14 | – | 13 | 1 | 1 | 2 | – | 1 | – | – | – |
|  | Cum. % | – | – | 8.8 | – | 12.3 | – | 14.0 | – | 43.9 | – | 68.4 | – | 91.2 | 93.0 | 94.7 | 98.2 | – | 100 | – | – | – |
|  | % | – | – | 8.8 | – | 3.5 | – | 1.8 | – | 29.8 | – | 24.6 | – | 22.8 | 1.8 | 1.8 | 3.5 | – | 1.8 | – | – | – |
| CLR | N | – | 10 | – | – | 8 | – | – | – | 1 | – | 2 | – | 1 | 10 | 10 | – | – | 3 | – | – | 12 |
|  | Cum. % | – | 17.5 | – | – | 31.6 | – | – | – | 33.3 | – | 36.8 | – | 38.6 | 56.1 | 73.7 | – | – | 78.9 | – | – | 100 |
|  | % | – | 17.5 | – | – | 14.0 | – | – | – | 1.8 | – | 3.5 | – | 1.8 | 17.5 | 17.5 | – | – | 5.3 | – | – | 21.1 |
| DOX | N | – | – | – | – | 3 | – | 14 | – | 1 | – | – | – | – | 2 | 6 | 8 | 23 | – | – | – | – |
|  | Cum. % | – | – | – | – | 5.3 | – | 29.8 | – | 31.6 | – | – | – | – | 35.1 | 45.6 | 59.6 | 100 | – | – | – | – |
|  | % | – | – | – | – | 5.3 | – | 24.6 | – | 1.8 | – | – | – | – | 3.5 | 10.5 | 14.0 | 40.4 | – | – | – | – |
| ERY | N | – | 3 | – | – | 14 | – | 1 | – | – | – | 1 | – | 2 | 1 | 11 | 8 | – | 1 | – | – | 15 |
|  | Cum. % | – | 5.3 | – | – | 29.8 | – | 31.6 | – | – | – | 33.3 | – | 36.8 | 38.6 | 57.9 | 71.9 | – | 73.7 | – | – | 100 |
|  | % | – | 5.3 | – | – | 24.6 | – | 1.8 | – | – | – | 1.8 | – | 3.5 | 1.8 | 19.3 | 14.0 | – | 1.8 | – | – | 26.3 |
| LVX | N | – | – | – | – | – | – | – | 1 | – | – | – | – | 13 | 39 | 2 | – | – | 1 | 1 | – | – |
|  | Cum. % | – | – | – | – | – | – | – | 1.8 | – | – | – | – | 24.6 | 93.0 | 96.5 | – | – | 98.2 | 100 | – | – |
|  | % | – | – | – | – | – | – | – | 1.8 | – | – | – | – | 22.8 | 68.4 | 3.5 | – | – | 1.8 | 1.8 | – | – |
| MXF | N | – | – | – | 2 | – | – | 27 | – | 25 | – | – | – | 1 | 1 | 1 | – | – | – | – | – | – |
|  | Cum. % | – | – | – | 3.5 | – | – | 50.9 | – | 94.7 | – | – | – | 96.5 | 98.2 | 100 | – | – | – | – | – | – |
|  | % | – | – | – | 3.5 | – | – | 47.4 | – | 43.9 | – | – | – | 1.8 | 1.8 | 1.8 | – | – | – | – | – | – |
| PEN | N | 3 | – | 5 | – | 3 | – | 5 | – | 17 | – | 14 | – | 6 | 1 | 2 | 1 | – | – | – | – | – |
|  | Cum. % | 5.3 | – | 14.0 | – | 19.3 | – | 28.1 | – | 57.9 | – | 82.5 | – | 93.0 | 94.7 | 98.2 | 100 | – | – | – | – | – |
|  | % | 5.3 | – | 8.8 | – | 5.3 | – | 8.8 | – | 29.8 | – | 24.6 | – | 10.5 | 1.8 | 3.5 | 1.8 | – | – | – | – | – |
| TET | N | – | – | – | 1 | – | – | – | – | 14 | – | 3 | – | – | 1 | – | 1 | 37 | – | – | – | – |
|  | Cum. % | – | – | – | 1.8 | – | – | – | – | 26.3 | – | 31.6 | – | – | 33.3 | – | 35.1 | 100 | – | – | – | – |
|  | % | – | – | – | 1.8 | – | – | – | – | 24.6 | – | 5.3 | – | – | 1.8 | – | 1.8 | 64.9 | – | – | – | – |
| SXT | N | – | – | – | – | – | 1 | – | – | – | – | 1 | – | 1 | 2 | 2 | 27 | – | 22 | 1 | – | – |
|  | Cum. % | – | – | – | – | – | 1.8 | – | – | – | – | 3.5 | – | 5.3 | 8.8 | 12.3 | 59.6 | – | 98.2 | 100 | – | – |
|  | % | – | – | – | – | – | 1.8 | – | – | – | – | 1.8 | – | 1.8 | 3.5 | 3.5 | 47.4 | – | 38.6 | 1.8 | – | – |

–, not applicable; AMC, amoxicillin/clavulanic acid; AMX, amoxicillin; AZM, azithromycin; CDR, cefdinir; CEC, cefaclor; CFM, cefixime; CLR, clarithromycin; CPD, cefpodoxime; CRO, ceftriaxone; CTB, ceftibuten; CTX, cefotaxime; Cum., cumulative; CXM, cefuroxime; DOX, doxycycline; ERY, erythromycin; LVX, levofloxacin; MXF, moxifloxacin; PEN, penicillin; SXT, trimethoprim/sulfamethoxazole; TET, tetracycline.

Bold vertical bars in table correspond to the CLSI-susceptible breakpoints.

**Supplementary Table 2.** MIC distribution data for *H. influenzae* isolates (*n* = 67) from Pakistan

|  |  | Number of isolates at MIC (mg/L) | | | | | | | | | | | | | | | | | | | | | | | | | | | |
| --- | --- | --- | --- | --- | --- | --- | --- | --- | --- | --- | --- | --- | --- | --- | --- | --- | --- | --- | --- | --- | --- | --- | --- | --- | --- | --- | --- | --- | --- |
| Antimicrobial |  | ≤0.001 | ≤0.002 | 0.002 | 0.004 | ≤0.008 | 0.008 | ≤0.015 | 0.015 | ≤0.03 | 0.03 | ≤0.06 | 0.06 | ≤0.12 | 0.12 | ≤0.25 | 0.25 | 0.5 | 1 | 2 | 4 | 8 | >8 | 16 | 32 | >32 | 64 | 128 |  |
| AMX | N | – | – | – | – | – | – | – | – | 1 | – | – | – | – | 4 | – | 26 | 12 | 8 | 13 | – | 1 | – | 1 | – | – | – | 1 |  |
|  | Cum. % | – | – | – | – | – | – | – | – | 1.5 | – | – | – | – | 7.5 | – | 46.3 | 64.2 | 76.1 | 95.5 | – | 97.0 | – | 98.5 | – | – | – | 100 |  |
|  | % | – | – | – | – | – | – | – | – | 1.5 | – | – | – | – | 6.0 | – | 38.8 | 17.9 | 11.9 | 19.4 | – | 1.5 | – | 1.5 | – | – | – | 1.5 |  |
| AMC (2:1) | N | – | – | – | – | – | – | – | – | – | – | – | – | – | 3 | – | 16 | 24 | 9 | 9 | 6 | – | – | – | – | – | – | – |  |
|  | Cum. % | – | – | – | – | – | – | – | – | – | – | – | – | – | 4.5 | – | 28.4 | 64.2 | 77.6 | 91.0 | 100 | – | – | – | – | – | – | – |  |
|  | % | – | – | – | – | – | – | – | – | – | – | – | – | – | 4.5 | – | 23.9 | 35.8 | 13.4 | 13.4 | 9.0 | – | – | – | – | – | – | – |  |
| AMC  [2 mg/L] | N | – | – | – | – | – | – | – | – | 2 | – | – | 1 | – | 5 | – | 29 | 9 | 10 | 11 | – | – | – | – | – | – | – | – |  |
|  | Cum. % | – | – | – | – | – | – | – | – | 3.0 | – | – | 4.5 | – | 11.9 | – | 55.2 | 68.7 | 83.6 | 100 | – | – | – | – | – | – | – | – |  |
|  | % | – | – | – | – | – | – | – | – | 3.0 | – | – | 1.5 | – | 7.5 | – | 43.3 | 13.4 | 14.9 | 16.4 | – | – | – | – | – | – | – | – |  |
| AMP | N | – | – | – | – | – | – | – | – | 3 | – | – | 2 | – | 26 | – | 10 | 5 | 16 | 2 | 1 | – | – | 1 | – | – | 1 | – |  |
|  | Cum. % | – | – | – | – | – | – | – | – | 4.5 | – | – | 7.5 | – | 46.3 | – | 61.2 | 68.7 | 92.5 | 95.5 | 97.0 | – | – | 98.5 | – | – | 100 | – |  |
|  | % | – | – | – | – | – | – | – | – | 4.5 | – | – | 3.0 | – | 38.8 | – | 14.9 | 7.5 | 23.9 | 3.0 | 1.5 | – | – | 1.5 | – | – | 1.5 | – |  |
| AZM | N | – | – | – | – | – | – | – | – | – | – | – | – | 3 | – | – | 5 | 27 | 22 | 9 | – | – | 1 | – | – | – | – | – |  |
|  | Cum. % | – | – | – | – | – | – | – | – | – | – | – | – | 4.5 | – | – | 11.9 | 52.2 | 85.1 | 98.5 | – | – | 100 | – | – | – | – | – |  |
|  | % | – | – | – | – | – | – | – | – | – | – | – | – | 4.5 | – | – | 7.5 | 40.3 | 32.8 | 13.4 | – | – | 1.5 | – | – | – | – | – |  |
| CEC | N | – | – | – | – | – | – | – | – | – | – | – | – | – | – | 2 | – | 10 | 21 | 4 | 11 | 12 | – | 4 | 3 | – | – | – |  |
|  | Cum. % | – | – | – | – | – | – | – | – | – | – | – | – | – | – | 3.0 | – | 17.9 | 49.3 | 55.2 | 71.6 | 89.6 | – | 95.5 | 100 | – | – | – |  |
|  | % | – | – | – | – | – | – | – | – | – | – | – | – | – | – | 3.0 | – | 14.9 | 31.3 | 6.0 | 16.4 | 17.9 | – | 6.0 | 4.5 | – | – | – |  |
| CDR | N | – | – | – | – | – | – | – | – | – | – | 4 | – | – | 23 | – | 19 | 10 | 5 | 6 | – | – | – | – | – | – | – | – |  |
|  | Cum. % | – | – | – | – | – | – | – | – | – | – | 6.0 | – | – | 40.3 | – | 68.7 | 83.6 | 91.0 | 100 | – | – | – | – | – | – | – | – |  |
|  | % | – | – | – | – | – | – | – | – | – | – | 6.0 | – | – | 34.3 | – | 28.4 | 14.9 | 7.5 | 9.0 | – | – | – | – | – | – | – | – |  |
| CFM | N | – | – | – | – | 5 | – | – | 20 | – | 29 | – | 5 | – | 3 | – | 2 | 2 | 1 | – | – | – | – | – | – | – | – | – |  |
|  | Cum. % | – | – | – | – | 7.5 | – | – | 37.3 | – | 80.6 | – | 88.1 | – | 92.5 | – | 95.5 | 98.5 | 100 | – | – | – | – | – | – | – | – | – |  |
|  | % | – | – | – | – | 7.5 | – | – | 29.9 | – | 43.3 | – | 7.5 | – | 4.5 | – | 3.0 | 3.0 | 1.5 | – | – | – | – | – | – | – | – | – |  |
| CTX | N | – | 15 | – | 8 | – | 13 | – | 11 | – | 8 | – | 5 | – | 6 | – | 1 | – | – | – | – | – | – | – | – | – | – | – |  |
|  | Cum. % | – | 22.4 | – | 34.3 | – | 53.7 | – | 70.1 | – | 82.1 | – | 89.6 | – | 98.5 | – | 100 | – | – | – | – | – | – | – | – | – | – | – |  |
|  | % | – | 22.4 | – | 11.9 | – | 19.4 | – | 16.4 | – | 11.9 | – | 7.5 | – | 9.0 | – | 1.5 | – | – | – | – | – | – | – | – | – | – | – |  |
| CPD | N | – | – | – | – | – | – | 8 | – | – | 23 | – | 10 | – | 12 | – | 7 | 4 | 2 | 1 | – | – | – | – | – | – | – | – |  |
|  | Cum. % | – | – | – | – | – | – | 11.9 | – | – | 46.3 | – | 61.2 | – | 79.1 | – | 89.6 | 95.5 | 98.5 | 100 | – | – | – | – | – | – | – | – |  |
|  | % | – | – | – | – | – | – | 11.9 | – | – | 34.3 | – | 14.9 | – | 17.9 | – | 10.4 | 6.0 | 3.0 | 1.5 | – | – | – | – | – | – | – | – |  |
| CTB | N | – | – | – | – | – | – | – | 4 | – | 24 | – | 10 | – | 8 | – | 9 | 4 | 5 | 2 | 1 | – | – | – | – | – | – | – |  |
|  | Cum. % | – | – | – | – | – | – | – | 6.0 | – | 41.8 | – | 56.7 | – | 68.7 | – | 82.1 | 88.1 | 95.5 | 98.5 | 100 | – | – | – | – | – | – | – |  |
|  | % | – | – | – | – | – | – | – | 6.0 | – | 35.8 | – | 14.9 | – | 11.9 | – | 13.4 | 6.0 | 7.5 | 3.0 | 1.5 | – | – | – | – | – | – | – |  |
| CRO | N | 8 | – | 20 | 14 | – | 7 | – | 10 | – | 3 | – | 5 | – | – | – | – | – | – | – | – | – | – | – | – | – | – | – |  |
|  | Cum. % | 11.9 | – | 41.8 | 62.7 | – | 73.1 | – | 88.1 | – | 92.5 | – | 100 | – | – | – | – | – | – | – | – | – | – | – | – | – | – | – |  |
|  | % | 11.9 | – | 29.9 | 20.9 | – | 10.4 | – | 14.9 | – | 4.5 | – | 7.5 | – | – | – | – | – | – | – | – | – | – | – | – | – | – | – |  |
| CXM | N | – | – | – | – | – | – | – | – | – | – | – | 1 | – | 2 | – | 14 | 24 | 4 | 14 | 5 | 1 | – | 2 | – | – | – | – |  |
|  | Cum. % | – | – | – | – | – | – | – | – | – | – | – | 1.5 | – | 4.5 | – | 25.4 | 61.2 | 67.2 | 88.1 | 95.5 | 97.0 | – | 100 | – | – | – | – |  |
|  | % | – | – | – | – | – | – | – | – | – | – | – | 1.5 | – | 3.0 | – | 20.9 | 35.8 | 6.0 | 20.9 | 7.5 | 1.5 | – | 3.0 | – | – | – | – |  |
| CLR | N | – | – | – | – | – | – | – | – | – | – | – | – | – | – | 2 | – | 2 | – | 13 | 30 | 16 | – | 3 | – | 1 | – | – |  |
|  | Cum. % | – | – | – | – | – | – | – | – | – | – | – | – | – | – | 3.0 | – | 6.0 | – | 25.4 | 70.1 | 94.0 | – | 98.5 | – | 100 | – | – |  |
|  | % | – | – | – | – | – | – | – | – | – | – | – | – | – | – | 3.0 | – | 3.0 | – | 19.4 | 44.8 | 23.9 | – | 4.5 | – | 1.5 | – | – |  |
| LVX | N | – | – | – | – | – | 6 | – | 23 | – | 2 | – | 6 | – | 1 | – | – | 14 | 3 | 2 | – | – | 10 | – | – | – | – | – |  |
|  | Cum. % | – | – | – | – | – | 9.0 | – | 43.3 | – | 46.3 | – | 55.2 | – | 56.7 | – | – | 77.6 | 82.1 | 85.1 | – | – | 100 | – | – | – | – | – |  |
|  | % | – | – | – | – | – | 9.0 | – | 34.3 | – | 3.0 | – | 9.0 | – | 1.5 | – | – | 20.9 | 4.5 | 3.0 | – | – | 14.9 | – | – | – | – | – |  |
| MXF | N | – | – | – | – | – | 9 | – | 18 | – | 5 | – | 5 | – | 1 | – | 2 | 13 | 3 | – | 1 | 7 | 3 | – | – | – | – | – |  |
|  | Cum. % | – | – | – | – | – | 13.4 | – | 40.3 | – | 47.8 | – | 55.2 | – | 56.7 | – | 59.7 | 79.1 | 83.6 | – | 85.1 | 95.5 | 100 | – | – | – | – | – |  |
|  | % | – | – | – | – | – | 13.4 | – | 26.9 | – | 7.5 | – | 7.5 | – | 1.5 | – | 3.0 | 19.4 | 4.5 | – | 1.5 | 10.4 | 4.5 | – | – | – | – | – |  |
| TET | N | – | – | – | – | – | – | – | – | – | – | – | – | 6 | – | – | 49 | 8 | 1 | – | – | 1 | – | 2 | – | – | – | – |  |
|  | Cum. % | – | – | – | – | – | – | – | – | – | – | – | – | 9.0 | – | – | 82.1 | 94.0 | 95.5 | – | – | 97.0 | – | 100 | – | – | – | – |  |
|  | % | – | – | – | – | – | – | – | – | – | – | – | – | 9.0 | – | – | 73.1 | 11.9 | 1.5 | – | – | 1.5 | – | 3.0 | – | – | – | – |  |
| SXT | N | – | – | – | – | 1 | – | – | – | – | – | – | 2 | – | 2 | – | 3 | 1 | 1 | – | 7 | 18 | 32 | – | – | – | – | – |  |
|  | Cum. % | – | – | – | – | 1.5 | – | – | – | – | – | – | 4.5 | – | 7.5 | – | 11.9 | 13.4 | 14.9 | – | 25.4 | 52.2 | 100 | – | – | – | – | – |  |
|  | % | – | – | – | – | 1.5 | – | – | – | – | – | – | 3.0 | – | 3.0 | – | 4.5 | 1.5 | 1.5 | – | 10.4 | 26.9 | 47.8 | – | – | – | – | – |  |

–, not applicable; AMC, amoxicillin/clavulanic acid; AMP, ampicillin; AMX, amoxicillin; AZM, azithromycin; CDR, cefdinir; CEC, cefaclor; CFM, cefixime; CLR, clarithromycin; CPD, cefpodoxime; CRO, ceftriaxone; CTB, ceftibuten; CTX, cefotaxime; Cum., cumulative; CXM, cefuroxime; LVX, levofloxacin; MXF, moxifloxacin; SXT, trimethoprim/sulfamethoxazole; TET, tetracycline.

Bold vertical bars in table correspond to the CLSI-susceptible breakpoints.
